# Proceedings of Montgomery Co., Ill., Medical Society

**Published:** 1873-07-15

**Authors:** Thos. D. Washburn

**Affiliations:** Secretary; Litchfield, Ill.


### PROCEEDINGS OF MONTGOMERY COUNTY MEDICAL SOCIETY.

LITCHFIELD, Ill., June 23, 1873.

The Society met at the office of Drs. Colt & Blackwelder, in Litchfield, on the 23d ult., at 9 o'clock A. M., the President, Dr. H. H. Hood, in the chair. The minutes of the last meeting were read and adopted. The roll called, a good attendance being present. The presentation of written reports being next in order, Dr. I. W. Fink read an interesting paper on some of the graver forms of disease, dwelling particularly on peritoneal puerperal and erysipelatous cases, giving statistics of mortality, and the months which were the most fatal. The pathology of the latter was made prominent as an index to treatment. Free and prompt incisions were recommended in the phlegmonous form. The paper elicited a spirited discussion, and a variety of views were expressed, both as to pathology and treatment. Dr. Barton was inclined to favor the use of cold, in peritoneal cases, freely and continuously applied, so long as agreeable to the patient—believing the best results flowed from it, in combination with antiphlogistic treatment and liberal use of opium.

Dr. Harman confirmed this practice, and reported several cases of a bad type, which he believed were indebted to this for their recovery. He proceeded to draw a comparison between this and the use of warm fomentations, giving a decided preference to the former.

In regard to *local* applications, in cases of erysipelas, he he had some doubts as to the *amount* of utility in them; believing the disease a blood poison, and that the treatment should be largely constitutional, and generally of a supporting character.

Dr. Cook thought he had received much benefit from the local use of the tinct. iodine, diluted with a persevering and thorough course of tonics—iron and quinine having the prominence.

Dr. Washburn's experience agreed with Drs. Barton and Harmon as to the use of cold in peritonitis, beginning with water of ordinary temperature, and adding ice when agreeable to the patient—continuing this course until the fever subsided, and the patient objected to its use. Besides the iodine as a local application, he had been in the habit of using glycerine and carbolic acid, especially on children, on account of its anæsthetic influence. Tincture of chloride ferri, saturated with quinine, he had found also serviceable, both externally and internally.

Reports of cases being called for, Dr. Washburn reported a case of protracted and severe Sciatica, the patient, Mr. Abner Short, being present. After exhausting *all* the usual treatment, among which was blistering and dressing the same with morphia, galvanism, aconite, iodide and bromide of potassa, alteratives and tonics, at the end of a month he was forced to use the hypodermic injection of morphia, the pain was so severe. In a few minutes afterwards the pain disappeared, and the effect continued for some twenty-four hours ; and since then, under the daily use of this for more than six weeks, he has left his bed, is going about, and gaining strength; but when the influence dies out the injection must be repeated. Query: How shall we *wean* him?

The discussion of this paper was postponed until 2 P. M. Dr. Colt having been called away, the report on Surgical Treatment of a Class of Female Diseases was postponed. No other reports being at hand, Dr. Washburn presented a brief paper on the subject recommended by the President of the last National Medical Association, Dr. Thos. M. Logan, viz.: "The Duty of Using the Public Press to Impart such Useful Medical Information as the People Need, and have a Just Right to Expect."

The Doctor, fully endorsing the President's views, and appealing to the profession to lay aside all false notions and unsafe precedents, believed the time had arrived when we should be heard through the daily press in our efforts to educate the people in many things connected with Physiology, Hygiene, and the great science of life.

Th paper drew out a lively discussion as to the manner, extent, and propriety of the same, but was generally endorsed, the debate extending past noon. The Secretary announced an invitation to the Society from Dr. and Mrs. Hood, to tea at 6 P. M., which, on motion, was accepted.

Adjourned to 1½ P. M.

AFTERNOON SESSION.

The Society met at 1½ P. M. Dr. Caldwell, of Zanesville, put in an appearance, and, on motion of the Censors, was admitted a member of the Society.

Dr. Cook read a case of Cerebro-Spinal Meningitis, followed by another paper by Dr. Hood on a case of injury complicated with Cerebro-Spinal Meningitis, and reports on the various forms which this disease assumes. Its pathology and treatment were ably discussed by Drs. Caldwell, Harmon, Barton, Fink, and others. The results of treatment in the late epidemic, though of greater severity, were far more favorable than any *previous*, and the rate of mortality largely reduced. Of fifteen cases treated by Dr. Caldwell, only four proved fatal. Ergot, cantharides, and calabar bean, were prominent remedial agents.

At the close of this, a large interchange of views was had, and, on motion, a committee was appointed to collect statistics and report at our annual meeting. The President named Dr. I. W. Fink as chairman, and all the members were invited to forward to him everything of interest connected with this very grave disease.

The case of Sciatica being again called up, Dr. Cook suggested the hypodermic treatment be continued, and the patient be put on a nerve tonic, like the compound phosphorous pill, or ioduform and iron. Dr. Caldwell advised the withdrawal of the opiate as soon as possible, some milder anodyne, and more liberal use of galvanism—the continuous current for four or five hours every day—dry cupping, etc., with the use of tonics. Dr. Colt suggested vapor baths and counter-irritants, and, if necessary, a seton. Some had found benefit from strychnia, belladonna and stramonium combined.

The hour having arrived, the Society took a recess to witness the removal of a steatomatous tumor by Dr. Colt, at the Bowlby House, from a married lady, otherwise healthy. This tumor had been observed for over two years, and occasioned much pain, owing to its peculiar position in the region of the occiput, and being somewhat imbedded among the tendons and fascia, required some time and patience to remove. The Dr., placing his patient under the influence of ether and chloroform, made as small an incision as was possible, so as to avoid an unpleasant scar, and gradually dissected it all out. Immediately following this, a boy of some ten years of age being present, with congenital club-foot, he was etherized, and tendo-achilis and some of the fascia and tendons on the inner sole of the foot severed by the subcutaneous operation, giving the Society a little variety from their protracted labors on a very hot day. Three other patients were also present for examination and advice—two of Dr. Whitten's. A case of paralysis of fore-arm, the young man having been thrown from a wagon and the wheel passing over it just above the elbow, making some abrasion of the skin only. He was unable to raise the hand or arm, though he had a fair grip in his fingers. Strong liniments, the douche, and galvanism, with strychnia internally, had been used for some weeks, but very little relief given. The other, a case of amaurosis from a blow. No perceptible difference from the sound eye (Referred to specialists). Third, Mr. ——, some fifty years of age, sallow, thin and cyanotic in appearance; had been an invalid for months, and had suffered much from many physicians; circulation not disturbed; some induration of right epigastrium and over the liver, with quite a jaundiced appearance of abdomen, while one inch to the right, and two inches above umbilicus, there was evident softening and *pointing*, and the conviction was *abscess*—no evidence of *embolism*.

Dr. Hood presented a very singular case of acute rheumatism in a girl of thirteen, having had a recurrence of the disease annually since she was six years of age, and recovering without unpleasant sequelæ. Query: How can its recurrence be prevented?

The following resolutions were then acted upon:

1. *Resolved*, That the organization of a *District Medical Society*, comprising within its limits such counties as are of easy access to each other, would be promotive of medical science and the best interests of the profession participating therein, and of the community served by them.

2. *Resolved*, That, with a view to the organization of such a Society, the Secretary of this Association be instructed to open correspondence with the Medical Societies of the counties of Sangamon, Macon, Christian, Shelby, Madison, Macoupin, Bond and Fayette.

Which were unanimously passed.

3. *Resolved*, That as members of the Medical profession, we ask no *class* legislation; we foster no isms or pathy, but we protest against these *traveling* mountebanks, who swindle the people with false pretenses. We think the time has arrived when legislation should carry out the *will* of the people by declaring them nuisances, and every municipality should impose such onerous license as would insure prohibition. We ask that the public be protected against regular and irregular quackery, and every one who administers and attempts to practice medicine be required to give evidence of adequate knowledge of the system and the remedy that every bottle and package of medicine exposed for sale be labeled with the ingredients of the same, and that the vile *things* that pander to the corrupt and the vicious, and publicly advertise to destroy life, and prevent the laws of nature, should be dealt with as the grossest and most abandoned criminals of the age.

Action on this resolution was laid over to the annual meeting of the Society in December next; and, on motion of Dr. Barton, the Secretary was instructed to correspond with the local and district societies of the State in regard to their co-operation in the action which the resolution contemplates.

On motion, the Society adjourned, when the members in a body proceeded to Dr. Hood's and partook of a bountiful repast, thus closing one of the most profitable and pleasant Society meetings we have ever enjoyed.

THOS. D. WASHBURN,
*Secretary.*

